# Transcranial cerebral oximetry in newborn infants on mechanical ventilation as a method for prevention of hyperoxia and oxidative stress

**DOI:** 10.1186/cc10900

**Published:** 2012-03-20

**Authors:** V Estrin, A Simonova

**Affiliations:** 1 Scientific Research Institute of Obstetrics and Pediatrics, Rostov on Don, Russia

## Introduction

The aim of this research was the effectiveness of respiratory therapy in infants with respiratory distress syndrome (RDS) who are ventilated by correcting the oxygen status and parameter optimization of ventilation by determining the oxygen saturation in the brain by transcranial cerebral oximetry (TCO).

## Methods

A total of 24 infants born in the physiological department of a maternity hospital in RNIIAP was studied. All of the children were measured for the saturation of brain tissue oxygen (SctL, SctR) with a cerebral oximeter (ForeSight; USA) at 1, 3 and 5 days after birth. Later in the study, two groups of newborn infants on mechanical ventilation were included. Patients of group 1 (*n *= 38), modes of mechanical ventilation and FiO_2 _were determined under control TCO to bring rates of cerebral oxygenation to the age norm. Patients of group 2 (*n *= 37), mode selection and ventilator FiO_2 _were carried out under the control of pulse oximetry and partial oxygen tension (pO_2_) in acid-base balance, without regard for performance of TCO. In all patients were determined serum peroxides (Oxystat test; BIOMEDICA GRUPPE, Germany), as well as the oxidation products of proteins (AOPP) in the serum of a set of AOPP (Immunodiagnostik, USA) for 1, 5 and 10 days. We measured blood gas parameters with an automatic analyzer (ABL; Denmark).

## Results

Determined by the age norm, TCO indicators for healthy infants amounted in the left hemisphere of the brain to 79.2 ± 4.06% (*P *< 0.01), and in the right hemisphere to 84.89 ± 5.1% (*P *< 0.01). We established in the group of infants where the mode selection ventilation and FiO_2 _were carried out on the basis of TCO indicators, an average FiO_2 _in the inspired mixture of 21% with an average pO_2 _in blood capillaries 61.95 ± 20.16%, in contrast to FiO_2 _55% with pO_2 _-78.01 ± 18.93% in patients of group 2. Patients of group 1 showed significantly (in all cases *P *< 0.01) decreased length of stay on mechanical ventilation (from 9.4 to 5.6 bed-days), compared with the control group. Investigation of the activity markers of oxidative stress showed three times reduction of the oxidation products of proteins (AOPP) and twofold reduction of peroxides in patients in the study group, compared with the control group, to 10 days of observation (*P *< 0.05).

## Conclusion

Monitoring of oxygen saturation in the brain tissue by TCO in infants with RDS reduces the mortality rate and the term of mechanical ventilation and hyperoxia.

